# A prospective study of the impact of musculoskeletal pain and radiographic osteoarthritis on health related quality of life in community dwelling older people

**DOI:** 10.1186/1471-2474-13-168

**Published:** 2012-09-07

**Authors:** Laura L Laslett, Stephen J Quinn, Tania M Winzenberg, Kristy Sanderson, Flavia Cicuttini, Graeme Jones

**Affiliations:** 1 Menzies Research Institute Tasmania, University of Tasmania, Private Bag 23, Hobart, TAS, 7000, Australia; 2 Department of Epidemiology and Preventive Medicine, Monash University, Melbourne, Australia

**Keywords:** Quality of life, Ostearthritis, Knee, Osteoarthritis, Radiographic

## Abstract

**Background:**

Pain and radiographic changes are common in persons with osteoarthritis, but their relative contributions to quality of life are unknown.

**Methods:**

Prospective cohort study of 1098 men and women aged 50–80 years, randomly selected from the electoral roll. Participants were interviewed at baseline and approximately 2.6 and five years later. Participants self-reported prior diagnosis of arthritis and presence of joint pain. Joint space narrowing (JSN) and osteophytes at the hip and knee were assessed by X-ray. Quality of life (QoL) was assessed using the Assessment of QoL (AQoL) instrument. Data was analysed using linear regression and mixed modelling.

**Results:**

The median AQoL score at baseline was 7.0, indicating very good QoL. Prevalence of pain ranged from 38-62%. Over five years of observation, pain in the neck, shoulders, back, hips, hands, knees and feet were all independently and negatively associated with QoL, in a dose–response relationship. Diagnosed osteoarthritis at all sites was associated with poorer QoL but after adjustment for pain, this only remained significant at the back. Radiographic OA was not associated with QoL. While AQoL scores declined over five years, there was no evidence of an interaction between pain and time.

**Conclusions:**

Pain is common in older adults, is stable over time, and the strongest musculoskeletal correlate of QoL. It also mediates the association between diagnosed OA and QoL. Since the same factors were associated with quality of life over time as at baseline, this suggests that quality of life tracks over a five year period.

## Background

Quality of life (QoL) is a useful and widely-used measure of health status because it captures the personal and social context of patients’ lives in a quantifiable way, and predicts use of health care resources and mortality [1,2]. Osteoarthritis (OA) is a leading cause of disability amongst older adults, and persons with osteoarthritis typically score poorly on QoL measures. Aspects of QoL involving physical functioning and pain are the most affected, and patients who report pain typically report it at more than one site [3]. Number of sites of pain have been associated with increasing disability[3] and poorer overall health, sleep quality and psychological health [4]. However, it is unclear whether pain at different sites is additive in terms of effect on QoL. Radiographic markers of osteoarthritis are weakly associated with pain [5,6] but both are associated with poor QoL, and it is unclear if radiographic findings are independent of or a diagnosis of OA, or pain[7-9]. In addition, it is not known whether the cross-sectional associations track over time. Baseline back, knee and hip pain were associated with reducing QoL over four years of observation in a Chinese volunteer cohort[10] but this has not been reported in western populations, in other anatomical sites, or in a population which also has radiographic measures.

The aim of this study was to describe the association between osteoarthritis and QoL in a community dwelling population-based sample of older people over five years.

## Methods

### Participants

The Tasmanian Older Adult Cohort (TASOAC) is an ongoing, prospective, population-based study examining the determinants of osteoarthritis and osteoporosis in older community dwelling adults. Men and women aged 50–80 years in 2002 were selected from the electoral roll in Southern Tasmania (population 229,000) using sex-stratified simple random sampling without replacement (response rate 57%). Participants were excluded if they resided in an aged care facility. The research was approved by the Southern Tasmanian Health and Medical Human Research Ethics Committee and written informed consent was obtained from all participants. Participants attended clinic and completed questionnaires. Data collection included blood sampling, magnetic resonance (MR) imaging (not reported in this study), knee and hip X-ray and other correlates of knee and hip OA and osteoporosis. Baseline data (Phase 1) was collected from February 2002 to September 2004. Follow up data (Phase 2 and 3) was collected on average 2.6 (range 1.4 to 4.8) and 5 years (range 3.6 to 6.9 years) later. Participants who did not have an MRI at Phase 1 (n = 105) were excluded from further participation in the study, as TASOAC aimed to measure osteoarthritis progression.

### Quality of life

Health-related QoL was measured using the Assessment of Quality of Life (AQoL) questionnaire [11]. This is a generic QoL instrument with five subscales (Illness, Independent Living, Social Relationships, Physical Senses and Psychological Well-being, Table 1), each with three items with four response levels (scored 0–3 for each item). The AQoL is a valid measure of QoL[12] and is reliable in population-based settings (Cronbach’s α = 0.81) [13]. The AQoL was used as an unweighted, psychometric instrument providing ‘value’ profiles, rather than using the utility measures [11] such as the AQoL-4D. These use only four of the subscales, excluding the Illness subscale which includes questions about the use and reliance on prescribed medicines or medical aids and requirement for regular medical treatment, all of which are likely to be increased by pain or a diagnosis of OA. Total scores for each subscale therefore ranged from 0–9 and the total instrument 0–45, with higher scores in each scale indicating worse QoL.

### Physician diagnosed osteoarthritis, pain and rheumatoid arthritis

Participants completed questionnaires (n = 1099) which asked “Have you had been told by a doctor that you have osteoarthritis at any of these sites”, and “Do you experience pain at any of these sites?". The seven anatomical sites were neck, back, hands, shoulders, hips, knees, and feet. Participants were given the choice between answering "yes" or "no". Participants were also asked “Have you been told by a doctor that you have rheumatoid arthritis?” (yes/no). Questions were asked about pain at Phase 1, 2 and 3; doctor diagnosed OA at Phase 1 and 2, and about doctor diagnosed RA at Phase 1.

### X-ray

Participants had X-rays of both hips (n = 1014) and knees (n = 1020) in the standing anterio-posterior (AP) position at baseline only. Knee X-rays were taken of both knees with 15° of fixed knee flexion, and pelvic radiographs with both feet in 10° internal rotation. Films were scored individually for osteophytes and joint space narrowing (JSN) on a scale of 0–3 (where 0 = no disease and 3 = most severe disease) according to the Osteoarthritis Research Society International (OARSI) atlas [14] as previously described [15]. Hips and knees with scores 1–3 at any site were classified as having JSN or osteophytes. Two readers simultaneously assessed radiographs with immediate reference to the atlas. Scores for each participant were determined by consensus. Intraobserver repeatability was assessed in 40 participants (intraclass correlation coefficients (ICCs) 0.65 to 0.85 for the knee and 0.60– 0.87 for the hip) [15].

### Other factors

Leg strength (n = 1038) was measured to the nearest kilogram in both legs simultaneously, using a dynamometer (TTM Muscular Meter, Tokyo, Japan) as described in Scott, 2009a.[16] This tests isometric strength, predominantly of the quadriceps and hip extensors. Weight was measured to the nearest 0.1 kg (with shoes, socks, bulky clothing and headwear removed) using a single pair of calibrated electronic scales (Seca Delta Model 707). Height was measured to the nearest 0.1 cm (barefoot) using a stadiometer. Body mass index (BMI) was calculated [weight (kg)/(height (m)) ^2^. Physical activity levels were determined using pedometers (Omron HJ-003 & HJ-102; Omron Healthcare, Kyoto, Japan) as previously described [17]. Briefly, number of steps per day is an average of seven consecutive days and averaged across two time points in different seasons. We collected self-reported estimates of current cigarette smoking prevalence by questionnaire.

### Data analysis

We used Stata 10.0 (StataCorp LP) for statistical analyses. Statistical significance was set as a p value ≤0.05 (two-tailed). Sample characteristics were analysed using t-tests and chi-square tests as appropriate. Baseline data was analysed using multiple linear regression. Analyses were first adjusted for age, sex and body mass index (BMI) (Step 1); variables which demonstrated a statistically significant association with total AQoL score were put into the next analysis, with the confounders leg strength and RA. The purpose of this was to determine whether each factor was independently associated with QoL or whether they were no longer significant after adjusting for other factors, suggesting mediation of effect.

Multilevel mixed-effects linear regression were used for longitudinal analyses, clustering on ID, and adjusted for change in BMI and age over time, as these terms were statistically significant. These were intent to treat analyses and used all available data.

We transformed the total AQoL score using a square root transformation in order to meet the residual assumptions underlying linear regression. Regression coefficients were back-transformed, and the β value was reported for each dependent variable, calculated with all other continuous variables centred at their mean, and dichotomous variables with the reference group having a value of zero. As a sensitivity analysis, we re-ran models in Table 2 without the psychological wellbeing scale to assess the possible effects of psychological distress as an unmeasured confounder of QoL.

**Table 1:** **Assessment of Quality of Life (AQoL)**^**†**^**subscales at baseline: mean scores and range**

	**Mean (sd) n = 1098**	**Median**	**Range**
Illness	3.2 (2.6)	3	0 – 9
Independent Living	0.3 (0.9)	0	0 – 7
Relationships	0.7 (1.0)	0	0 – 8
Physical senses	0.9 (1.0)	1	0 – 5
Psychological wellbeing	2.3 (1.6)	2	0 – 9
Total AQoL score	7.4 (4.9)	7	0 – 29

^†^Higher scores indicate poorer QoL.

Distribution was skewed and kurtotic, hence analyses are transformed using a square root transformation, with back transformed results presented.

## Results

### Participants

A total of 1098 people (51% female, mean age 63.0 years) completed baseline questionnaires. Of the 993 participants with complete MRI data at Phase 1 and were therefore invited to return for Phase 2, 875 completed Phase 2 and 768 completed Phase 3. Participants who failed to complete Phase 2 or 3 (including those who did not have baseline MR imaging), were older, had higher BMI and pain at more sites at baseline than those who remained in the study.

### Characteristics of the study population at baseline

Table 2 displays the characteristics of the cohort at baseline, stratified by median AQoL score. Those with poorer QoL were older, had higher BMI, walked fewer steps per day, were more likely to be retired or receiving a disability pension and less likely to be employed; and more likely to have no formal educational qualifications (Table 2). They also had higher prevalence of diagnosed osteoarthritis (OA) and pain at all sites (Table 3). Diagnosis of RA and leg strength were also associated with QoL, as expected, (Table 2), and were adjusted for in final models. Pain at the anatomical regions of interest was common (prevalence 38-62%), with 87% of participants reporting pain in at least one joint. 8% of patients reported pain in all seven regions.

**Table 2:** Osteoarthritis correlates of total AQoL score at baseline, using linear regression

	**Prevalence %**	**Step 1: Multivariable *β *(95% CI) Adjusted for age, sex and BMI**^**f**^	**Step 2: Multivariable *β *(95% CI)**^**§ **^**Further adjusted for all variables significant in Step 1**
Diagnosed OA of:			
Neck	168 (15)	**2.72 (1.80 to 3.64)**	−0.32 (−0.96 to 0.32)
Shoulders	193 (18)	**3.58 (2.34 to 4.81)**	0.23 (−0.64 to 1.10)
Back	167 (15)	**3.41 (2.54 to 4.28)**	**0.71 (0.02 to 1.41)**
Hips	97 (9)	**3.05 (1.93 to 4.17)**	0.04 (−0.74 to 0.82)
Hands	113 (10)	**2.32 (1.41 to 3.23)**	0.09 (−0.55 to 0.72)
Knees	152 (14)	**2.48 (1.52 to 3.43)**	0.15 (−0.55 to 0.85)
Feet	103 (9)	**3.40 (2.21 to 4.59)**	0.30 (−0.49 to 1.09)
Hip JSN (yes/no)	377 (37)	0.34 (−0.31 to 0.99)	-
Knee JSN (yes/no)	688 (67)	0.06 (−0.60 to 0.72)	-
Hip osteophyte (yes/no)	190 (19)	−0.10 (−0.89 to 0.69)	-
Knee osteophyte (yes/no)	143 (14)	−0.31 (−1.21 to 0.59)	-
Pain in the:			
Neck (yes/no)	514 (47)	**3.14 (2.58 to 3.71)**	**0.65 (0.16 to 1.15)**
Shoulder (yes/no)	674 (62)	**3.35 (2.77 to 3.93)**	**1.03 (0.52 to 1.54)**
Back (yes/no)	481 (44)	**2.94 (2.39 to 3.50)**	**0.58 (0.12 to 1.05)**
Hip (yes/no)	481 (44)	**2.44 (1.83 to 3.04)**	0.26 (−0.18 to 0.70)
Hand (yes/no)	505 (46)	**2.63 (2.05 to 3.22)**	**0.50 (0.04 to 0.96)**
Knee (yes/no)	451 (41)	**2.72 (2.13 to 3.31)**	0.41 (−0.04 to 0.86)
Foot (yes/no)	412 (38)	**3.27 (2.64 to 3.89)**	**1.13 (0.62 to 1.63)**

Statistically significant (p ≤ 0.05) results indicated in bold type.

^f^ after adjustment for age, sex and BMI.

^§^ further adjusted for diagnosis of RA, arthritis at all sites or pain at all sites and leg strength.

R^2^ for final model (Step 2) = 27%; R^2^ excluding pain is 13%; R^2^ pain alone = 23%.

**Table 3:** **Characteristics of the study population at baseline, by quality of life**^**†**^

	**QoL better than median Mean ± SEM n (%) n = 525**	**QoL at median or worse Mean ± SEM n (%) n = 573**	**p-value**
Age (years)	61.9 ± 0.3	64 ± 0.3	**<0.001**
Gender (% male)	267 (51)	246 (47)	0.18
BMI weight (cm)/(height (m))^2^	27.3 ± 0.2	28.4 ± 0.2	**<0.001**
Height (cm)	167.7 ± 0.4	166.3 ± 0.4	**0.006**
Weight (kg)	77 ± 0.6	78.7 ±0.6	0.06
Current smokers	64 (12)	67 (12)	0.78
Number of steps per day	10373.9 ± 158.3	8597.9 ±154.7	**<0.001**
Education Level			**<0.001**
No formal qualification	54 (10)	126 (22)	
School or Intermediate certificate	104 (20)	114 (20)	
Higher School or Leaving Certificate	114 (22)	107 (19)	
Trade/apprenticeship	59 (11)	78 (14)	
Certificate/diploma	122 (23)	95 (17)	
University degree or higher	72 (14)	52 (9)	
Current employment			
Employed/self-employed (full or part time)	264 (50)	168 (29)	**<0.001**
Retired	178 (34)	240 (42)	
Disability pension	4 (0.8)	69 (12)	
Doctor-diagnosed rheumatoid arthritis (%)	6	18	**<0.001**
Leg strength (kg)	101.6 ± 1.38	86.3 ± 1.4	**<0.001**

† QoL was not normal and hence dichotomized at the median.

Health-related QoL scores at baseline were skewed with a mean AQoL score of 7.4 (SD 4.9) and a median of 7.0 (range 0 to 29). Summary results for individual subscales are shown in Table 1.

### Correlates of quality of life at baseline: cross-sectional analysis

Since presence of pain at the various sites was not strongly collinear, (Pearson’s correlation r= 0.21 – 0.51), individual sites were entered into the model separately.

Table 3 shows that physician diagnosis of OA at any of the sites was associated with poorer QoL after adjustment for age sex and BMI, but only physician diagnosed OA of the back remained significant after further adjustment for RA, diagnosed OA at other sites and pain. Radiographic OA of the hip or knee (JSN, osteophytes) were not associated with QoL in any analysis. Presence or absence of pain at five of the seven sites were independently associated with poor QoL after further adjustment for diagnosis of RA, leg strength, diagnosed OA and pain at other sites. Knee pain was of borderline statistical significance after adjustment for all correlates, p = 0.076), and hip pain was not significant.

**Figure 1:**
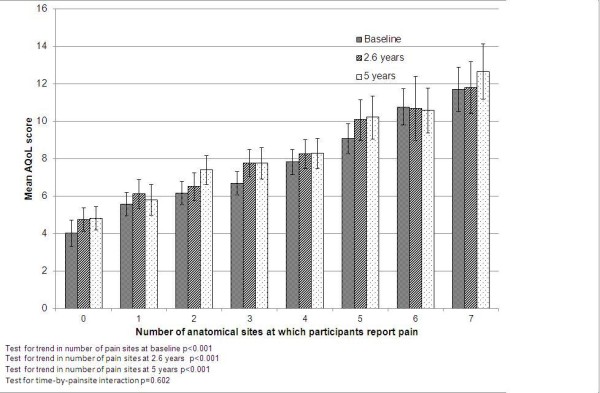
Mean total Assessment of Quality of Life (AQoL) score over time, by number of sites at which participants report pain and using multilevel mixed-effects linear regression.

The proportion of variance explained by the final model (R^2^, n = 1017) was 27%, of which 23% was explained by pain. There was also a strong linear association between the number of sites at which participants reported pain and QoL (Figure 1), suggesting a dose–response relationship. This association was significant at all three time points, and relatively constant over time (interaction p = 0.602).

We conducted sensitivity analyses without the psychological wellbeing subscale in order to assess whether the results from our total AQoL score were still valid after removing questions related to psychological factors. The same variables remained significant and coefficients were similar.

### Correlates of quality of life over time: Longitudinal analysis

Mean AQoL scores were 7.36 (95% CI 7.07 - 7.65) at Phase 1, 7.53 (95% CI 7.20 – 7.87) at Phase 2 and 7.82 (95% CI 7.47 – 8.17) by Phase 3. Average AQoL scores had significantly worsened by Phase 3 (p = 0.047), but not Phase 2 (0.44) using unadjusted data and unpaired t-tests. After adjusting for the changing composition of the sample over time using linear mixed models, reduction in means was significant at both Phase 2 and 3 (p < 0.001).

Table 4 shows a similar pattern of correlates of QoL to the analysis of correlates at baseline, although most effect sizes were smaller.

**Table 4:** Longitudinal analysis of arthritis correlates of total AQoL score over five years of follow up, using multilevel mixed-effects linear regression

	**Baseline to Phase 2 (2.6 year follow up)**		**Baseline to Phase 3 (5 year follow up)**	
	**Step 1: Multivariable β**	**Step 2: Multivariable β**	**Step 1: Multivariable β**	**Step 2: Multivariable β**
	**(adj. age sex BMI, change in BMI and age over time)**	**Adjusted further**^**¥**^	**(adj. age sex BMI, change in BMI and age over time)**	**Adjusted further**^**ϒ**^
Diagnosed OA of:				
Neck	**1.55 (0.96 to 2.15)**	0.01 (−0.5 to 0.52)		
Shoulders	**1.92 (1.22 to 2.61)**	0.37 (−0.23 to 0.96)		
Back	**1.76 (1.23 to 2.28)**	**0.59 (0.12 to 1.06)**		
Hips	**1.19 (0.54 to 1.84)**	−0.20 (−0.73 to 0.32)		
Hands	**1.35 (0.79 to 1.90)**	0.19 (−0.28 to 0.66)		
Knees	**1.50 (0.91 to 2.08)**	0.15 (−0.34 to 0.64)		
Feet	**1.40 (0.72 to 2.09)**	0.12 (−0.44 to 0.68)		
Presence or absence of pain in the:				
Neck	**1.79 (1.4 to 2.18)**	**0.55 (0.19 to 0.91)**	**1.20 (0.89 to 1.51)**	**0.42 (0.14 to 0.71)**
Shoulders	**1.80 (1.42 to 2.18)**	**0.66 (0.31 to 1.00)**	**1.36 (1.06 to 1.66)**	**0.64 (0.36 to 0.91)**
Back	**1.82 (1.45 to 2.19)**	**0.67 (0.33 to 1.00)**	**1.39 (1.09 to 1.68)**	**0.66 (0.39 to 0.94)**
Hips	**1.46 (1.07 to 1.85)**	**0.52 (0.19 to 0.85)**	**1.16 (0.85 to 1.47)**	**0.47 (0.20 to 0.74)**
Hands	**1.20 (0.82 to 1.59)**	0.19 (−0.13 to 0.51)	**0.91 (0.60 to 1.22)**	**0.27 (0.01 to 0.53)**
Knees	**1.51 (1.12 to 1.90)**	**0.43 (0.10 to 0.75)**	**1.10 (0.79 to 1.41)**	**0.44 (0.17 to 0.70)**
Feet	**0.98 (0.65 to 1.32)**	**0.36 (0.09 to 0.62)**	**0.75 (0.47 to 1.02)**	**0.26 (0.03 to 0.49)**

Statistically significant (p ≤ 0.05) results indicated in bold type.

^¥^ Results further adjusted for diagnosed OA at all sites, pain at all sites, presence of rheumatoid arthritis and leg strength.

^ϒ^Results further adjusted as for the analyses for Baseline to Phase 2, but without Diagnosed OA as this was not asked at Phase 3.

5 year follow up data includes data collected at Phase 1, 2 and 3 and is not limited to participants with complete data.

Radiographs not included as they were only collected at Phase 1.

After 2.6 years of observation, diagnosed OA (all sites) and presence or absence of pain (all sites) were significant. After further adjustment for the factors outlined above, diagnosed OA at the back remained significant as did pain at six of the seven anatomical sites. There were no significant interaction terms after adjustment for confounders and other covariates.

After five years of observation, pain at all sites was a significant independent determinant of QoL (Table 4). QoL amongst participants with neck pain remained stable whilst steadily worsening in those with no neck pain (p = 0.02 for interaction) but all other tests for interaction were not significant.

## Discussion

This population-based prospective study describes the contribution of multiple osteoarthritic correlates of QoL over five years of observation. Physician diagnosed OA of the back and pain at all sites were independent and stable correlates of QoL, and pain at multiple sites has an additive deleterious effect on QoL. With the exception of the back, pain appeared to mediate the association between diagnosed OA and QoL. Radiographic osteoarthritis was not associated with QoL.

In this study, the strongest musculoskeletal correlate of QoL was pain. Pain is a priority for patients seeking care [18] and thus it is perhaps not surprising that pain largely mediated the association between doctor diagnosed OA and QoL. Further, pain assessed at one site in cross sectional studies is known to be associated with poorer QoL, [19,20] but no studies that have looked at pain at many sites. Our data suggests that pain at all sites measured independently contribute to QoL, there is a dose response association between number of pain sites and QoL, and severity of pain is also related to QoL. Our data suggests that pain is very common in older adults in the community. Given that pain at individual joints and overall number of sites of joint pain were associated with poor QoL, this suggests that interventions to reduce the frequency and intensity of pain may be effective in improving QoL at the population level.

While there are some inconsistencies in the three analyses, the most weight should be put on the analysis over five years as it uses all the data and therefore is the most powerful. These results confirm and extend the findings of Woo *et al.*, 2009, [10] where pain at the back, hip (men only) and knees was associated with QoL over time in ethnic Chinese. Pain in the shoulders and back were the most important factors in our analyses, but knees, hips and even hands and feet were significant. The inconsistency with the hip may, in part, be due to patients have difficulty locating the correct anatomical position of the hips, [21] or that pain in the knee can actually be referred from the hip [22]. Knee pain was of borderline significance in cross-sectional analyses but became significant over time.

Diagnosed OA of the back was also an independent correlate of poor QoL (both in cross-sectional and longitudinal analyses), but diagnosed OA of the neck, shoulders, hips, hands, knees and feet were not once adjustment was made for the multiple sites of OA and for pain. This suggests that while pain mediates the associations between diagnosed OA and QoL at sites other than the back (neck, shoulders, hands, hips, knees and feet), the association between diagnosed OA of the back and QoL is only partially mediated by pain. It is well known that psychological factors such as depression are associated with chronic back pain but unfortunately we were not able to assess these in the current study.

There was no association between radiographic osteoarthritis and QoL at baseline, after adjusting for age, sex and BMI. This suggests that radiographic findings make no independent contribution to QoL, consistent with other studies which showed that the association between radiographic OA of the hand and function was largely mediated by pain, [23] and that pain is a better predictor of disability than radiographic change [24-26]. This differs from the findings of other studies [9,27], who found that radiographic OA was associated cross-sectionally with different disease-specific measure of QoL, after adjustment for pain and other covariates. Both of these measures of QoL had pain as a subscale, so this may explain why they found an association yet we did not. A strength of our study is that, unlike Norimatsu and colleagues, we have collected (self-reported) diagnosis of OA and radiographic findings separately (in addition to pain), and while finding them to be correlated, when both diagnosis and radiography appear together in one model, radiographic findings are no longer associated with QoL. Our data demonstrates that diagnosis of OA reflects more than radiographic evidence of joint damage, but that with the exception of diagnosed OA of the back, is not independent of pain.

Strengths of this study include the random population-based sampling and comprehensive data collection, and five-year period of observation, providing excellent external validity for our findings. Limitations include absence of information on psychological factors, such as diagnosed mental health conditions or psychological distress: this limits our ability to consider such conditions as covariates or effect modifiers, but our model is robust whether or not the mental health component of QoL is included, suggesting this is not a major issue. Additionally, the initial response rate of 57%, while lower than desirable, is similar to other comparable Australian studies, [19] and a lower response rate does not mean that relationships between outcome and exposure are necessarily biased [28]. Participants who did not continue in the study were older, heavier, with pain at more sites at baseline than the remaining participants. This should reduce the observed effect size of our findings, but since few associations were of borderline significance this should not have altered our conclusions. We did not seek to confirm doctor-diagnosed cases of arthritis, and therefore participants may have under-or over-reported diagnosed arthritis, and the extent to which this may have affected the findings of the study is unclear. However, use of self-reported doctor diagnosed OA appears to be a reasonable proxy for OA, as JSN was more common in participants reporting doctor-diagnosed OA at the hips and knees (hips OR 2.3, p < 0.001; knees OR 1.6, p = 0.023), and osteophytes more common in participants reporting knee (OR 4.10, p < 0.001), but not hip OA (OR 0.94, p = 0.83). We had X-rays only of the hips and knees, and so are not able to assess the association between ROA and QoL at other anatomic sites. However, unless the causal pathways at other sites are substantially different to those at the knees and hips, it is unlikely that radiographic OA at these sites would add any new information to the models.

## Conclusions

In conclusion, pain is the strongest musculoskeletal correlate of QoL, which has an additive deleterious effect on QoL, and mediates the effect of diagnosed OA (except in OA of the back). These associations are stable over time suggesting that pain has a consistent rather than an increasing deleterious effect. Since we found that the same factors were associated with quality of life over time as in the baseline analysis, this suggests that quality of life tracks over a five year period.

## Competing interests

The authors declared that they have no competing interests to declare.

## Author’s contributions

GJ designed the TASOAC study, the study from which the data in this manuscript is taken and formulated the hypotheses for this analysis. LLL carried out the statistical analysis with assistance from SJQ. TW KS and FC contributed to discussions about the underlying patterns of associations. LLL wrote the manuscript with assistance from GJ. All authors reviewed and critically revised the manuscript drafts, and read and approved the final manuscript.

## Authors’ information

The TASOAC study was supported by the National Health and Medical Research Council of Australia; Arthritis Foundation of Australia; Tasmanian Community Fund; Masonic Centenary Medical Research Foundation, Royal Hobart Hospital Research Foundation, and University of Tasmania Institutional Research Grants Scheme. Laura Laslett is supported by an Australian Government Australian Postgraduate Award. Graeme Jones is supported by a National Health and Medical Research Council practitioner fellowship. Tania Winzenberg is supported by an Osteoporosis Australia Australian and New Zealand Bone and Mineral Society/Amgen Fellowship. Kristy Sanderson is supported by an Australian Research Council Future Fellowship (FT0991524).

## Pre-publication history

The pre-publication history for this paper can be accessed here:

http://www.biomedcentral.com/1471-2474/13/168/prepub
